# Hoffa’s Osteochondroma - Para-articular Extrasynovial Infrapatellar Fat Pad Osteochondroma: A Case Report

**DOI:** 10.31729/jnma.6625

**Published:** 2021-08-31

**Authors:** Sunil Panta, Shrawan Kumar Thapa, Krishna Prasad Paudel, Manoj Kandel, Bishwa Raj Adhikari

**Affiliations:** 1 Department of Orthopedics, Bharatpur Hospital, Chitwan, Nepal

**Keywords:** *adipose tissue*, *knee joint*, *osteochondroma*

## Abstract

Osteochondroma usually arises from the metaphyseal region of growing bones. The occurrence of extraskeletal osteochondroma is rare with very few case reports. Para-articular osteochondroma is a type of extraskeletal osteochondroma. It frequently occurs around the knee, usually at infrapatellar Hoffa's fat pad. It is usually intracapsular but extrasynovial and arises from the capsule and connective tissues due to osteocartilaginous metaplasia. We present a case of 19-years male with anterior knee pain for 3 years, swelling, and deformity of the knee with flexion limitation for one year. Radiography revealed ovoid, corticated lesion free from adjoining bones. Mass interpreted as benign, so planned for excision. Well circumscribed nodule excised from the medial parapatellar approach. Histology revealed cartilaginous tissues surrounded by fibrous tissues with scattered enchondral ossification. Postoperatively and subsequent follow-up resulted in pain-free joint, complete recovery of range of motion with no clinicoradiological evidence of recurrence.

## INTRODUCTION

Osteochondroma is the most common benign bone tumor. It usually arises in the metaphyseal region of growing bones. Extraskeletal osteochondroma is very rare, manifestation is common in the knee particularly in the infrapatellar fat pad of Hoffa. It originates from the capsule or adjoining connective tissue as a result of osteocartilaginous metaplasia. It may be the sequel of Hoffa's disease which is the consequence of various traumatic events or repetitive microtrauma of the infrapatellar fat.^1-5^ We present a case of para-articular extrasynovial osteochondroma of the knee in a young adult and discuss the clinico-radiological features, histopathological features, pathogenesis, treatment, and outcome.

## CASE REPORT

A 19 years old male presented with gradually increasing swelling at the anterior aspect of the right knee with deformity, anterior knee pain for 3 years, and difficulty in flexing the knee for 1 year. The pain was primarily activity-related. No history of relevant trauma. There was no any mass in other parts of the body. On examination there was a non-tender, irregular, bony hard swelling prominent over the medial aspect of the patella tendon, flexion restricted beyond 90 degrees.

**Figure 1:**
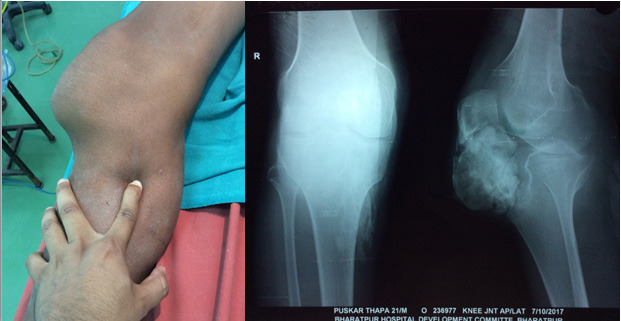
Preoperative clinical picture and radiograph.

Blood parameters were normal. Radiographs revealed a well-circumscribed, ovoid, corticated mass at the infrapatellar fat pad region. It was free from adjoining bones (Figure 1). Further radiological investigations not done due to financial constrain.

**Figure 2:**
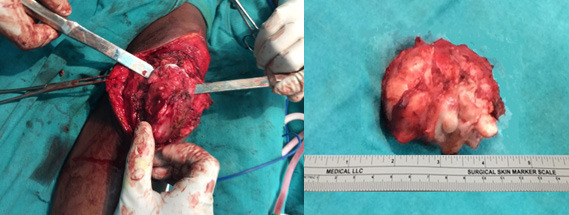
Intraoperative picture of marginal resection and excised mass.

Clinico-radiologically, it was interpreted as benign and marginal excision was planned. Well circumscribed, intracapsular, and extrasynovial mass measuring about (6x4x3) cm excised through the medial parapatellar approach (Figure 2).

**Figure 3:**
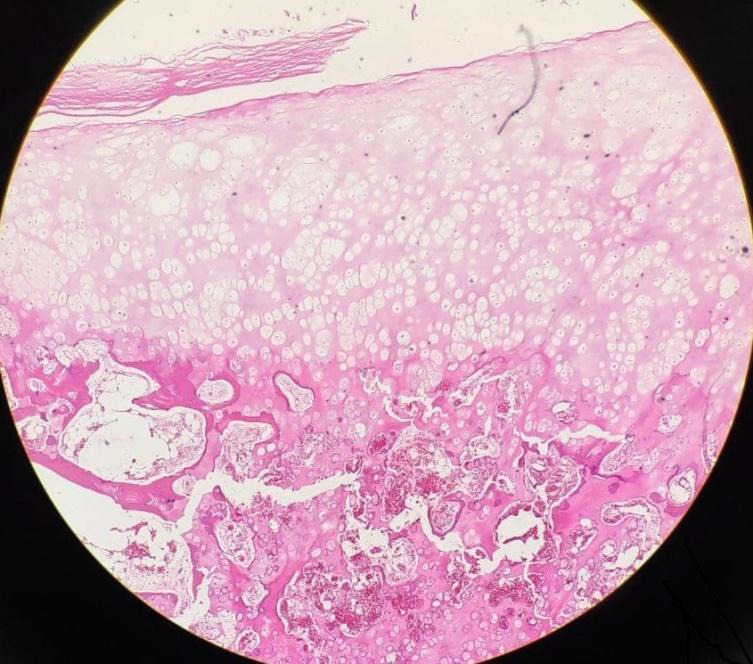
Histopathological picture showing cartilaginous tissue with scattered enchondral ossification and fibrous capsular tissue.

Histology revealed cartilaginous tissue with scattered enchondral ossification within, surrounded by capsular fibrous tissue. There was no evidence of malignant features (Figure 3). The pathological features were diagnostic of extraskeletal para-articular osteochondroma.

**Figure 4:**
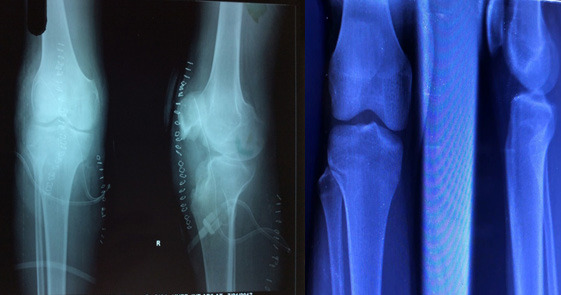
Immediate and 1 year follow-up radiographs.

Postoperatively knee mobilization and weight-bearing allowed as tolerated. Knee was pain-free, with complete recovery of range of motion on subsequent follow-up. There was no clinico-radiological evidence of recurrence at 1 year follow-up (Figure 4).

## DISCUSSION

Extraskeletal osteochondroma is very rare. There are 3 different types of extraskeletal osteochondroma: a) synovial chondromatosis, b) soft tissue chondroma and c) para-articular osteochondroma. In synovial chondromatosis, multiple intrasynovial osteocartilaginous nodules are seen as loose bodies. Soft tissue chondromas are usually seen at hands and feet. According to Reith et al, a lesion is called paraarticular osteochondroma if: (a) the lesion presents as a single, dominant mass, both radiographically and grossly; (b) the mass consists histopathologically both bone and cartilage, organized in a manner similar to conventional osteochondromas; and (c) the lesion is not intra-articular, that is, it does not arise within the synovial lining of a joint.^1-3^

Para-articular osteochondroma usually manifests in joints with a potential capsular space, such as the patellofemoral joint or infrapatellar Hoff's fat pad, although they may occur in other regions too.^1,4,5^ It may occur at any age, but most of them are reported at 3060 years.^1,6,7^ This is case of 19 years gentleman, with a solitary mass in the infrapatellar region lined by joint capsule externally and synovial membrane internally with no evidence of underlying bone continuity. Radiographs usually reveal a single, marginated bony mass with internal trabeculae. Histopathologically, mass is characterized by enchondral ossification surrounded by hyaline cartilage, fibrous and adipose tissue. So, the most appropriate term to define this lesion is "para-articular, extrasynovial osteochondroma". Similar clinico-radiological and histopathological findings are seen in other case reports on extraskeletal osteochondroma.^1-4,8-10^

The exact pathogenesis is still unknown, but it is supposed to be due to osteocartilaginous metaplasia of the articular or para-articular connective tissue, or metaplasia of Hoffa's fat pad due to trauma, or sequel of end-stage of Hoffa's disease (chronic impingement of Hoffa's fat pad). So, in this case, the pathogenesis is not relatable to a mechanical post-traumatic event or chronic impingement of Hoffa's fat pad but to primary osteocartilaginous metaplasia of infrapatellar _fat pad._^1,2,4,5^

CT scan and MRI were not done due to financial constrain, which is the limitation of this case report. Clinically and radiographically, the tumor was interpreted as benign, so we planned marginal resection and sent for histopathological examination which confirmed our diagnosis. Marginal resection is the treatment of choice, especially for para-articular osteochondroma.^2,3,6^ There is no known recurrence of the lesions after complete resection.^1,3,11^

Para-articular extrasynovial osteochondroma is an unusual lesion that commonly arises around the knee joint, particularly the infrapatellar Hoffa's fat pad due to the osteocartilaginous metaplasia of capsule and adjoining connective tissues. Clinico-radiological examinations are often diagnostic but integrated radio-pathological examination helps to distinguish it from other differentials. Marginal resection is the treatment of choice and these lesions are not known to recur.
